# Bridging the data latency gap: automated extraction of genomic biomarkers from unstructured clinical documents to support real-world oncology data

**DOI:** 10.1007/s10552-026-02252-y

**Published:** 2026-09-20

**Authors:** Qianyun Luo, Rui Zhang, Nikitha Vobugari, Jane Y. C. Hui, Schelomo Marmor

**Affiliations:** 1 University of Minnesota Medical School, Minneapolis, MN USA; 2 Division of Computational Health Sciences, Department of Surgery, University of Minnesota, Minneapolis, MN USA; 3 Division of Hematology, Oncology and Transplantation, Department of Medicine, University of Minnesota, Minneapolis, MN USA; 4 Division of Surgical Oncology, Department of Surgery, University of Minnesota, Minneapolis, MN USA; 5 Clinical Quality, Outcomes, Discovery and Evaluation Core (CQODE), University of Minnesota, Minneapolis, MN USA; 6 Center for Learning Health Systems Science (CLHSS), University of Minnesota, Minneapolis, MN USA; 7 Masonic Cancer Center, University of Minnesota, Minneapolis, MN USA

**Keywords:** Oncology, Genomics, Breast cancer, Optical character recognition

## Abstract

**Purpose:**

Real-world oncology data are essential for clinical research and precision cancer care. However, genomic biomarkers are often embedded in scanned, unstructured clinical documents requiring manual abstraction before becoming available in cancer registries, delaying real-world evidence generation. This study evaluated and compared three open-source optical character recognition (OCR) approaches, Tesseract, EasyOCR, and a hybrid implementation, to determine which best enables automated extraction of Oncotype DX recurrence scores and improves the timeliness and quality of real-world oncology data.

**Methods:**

We evaluated the feasibility of automated genomic data extraction using 675 Oncotype DX reports from a Midwestern U.S. health system. EasyOCR, Tesseract, and a hybrid OCR approach were used to extract recurrence scores from scanned reports. OCR-derived values were compared with manually abstracted scores and local cancer registry data. Performance was assessed using agreement, precision, recall, F1 score, and processing time. Multivariable logistic regression was performed to identify factors associated with discordance between registry-reported and manually abstracted scores.

**Results:**

The hybrid OCR approach demonstrated the highest performance, achieving 97% agreement with manual abstraction, precision of 0.997, recall of 0.972, and an F1 score of 0.984. Registry abstraction demonstrated comparable performance but required greater manual effort. Automated extraction substantially reduced processing time while maintaining high accuracy. Logistic regression showed registry discordance was largely independent of patient and tumor characteristics, with unknown progesterone receptor (PR) status as the only significant predictor.

**Conclusion:**

Automated extraction of genomic biomarkers represents a scalable approach to reducing delays in cancer data availability. Earlier capture of genomic information may support cancer registry modernization and improve real-world evidence generation in precision oncology.

## Introduction

Real-world data have become an increasingly important component of oncology research, supporting comparative effectiveness studies, quality improvement initiatives, precision medicine, and health policy development [1–6]. Cancer registries are a cornerstone of these efforts by providing standardized, population-based data that enable cancer surveillance, outcomes research, and evaluation of treatment patterns across health systems [7–10]. As genomic testing becomes integrated into routine cancer care, the timely availability of genomic biomarkers within registry databases is becoming increasingly important for both clinical research and real-world evidence generation.

Despite advances in registry infrastructure, genomic data remain one of the most difficult data elements to capture efficiently. Unlike structured laboratory results, many commercially available genomic assays are received as scanned Portable Document Format (PDF) reports or image-based documents that must be manually reviewed and abstracted before becoming available within cancer registries and research databases [11–15]. This manual workflow is labor intensive, susceptible to transcription errors, and often delays data availability by 12–18 months [11]. Consequently, genomic information that has already informed clinical decision-making may remain unavailable for population-level surveillance, quality improvement, and observational research long after patient care has occurred.

Reducing this data latency is increasingly important as learning health systems seek to generate near real-time evidence from routine clinical practice [14]. Timely access to structured genomic biomarkers enables more rapid evaluation of treatment patterns, supports quality measurement, and facilitates the development of artificial intelligence (AI) and machine learning applications using up-to-date oncology data [5, 6, 15]. Rather than replacing existing registry workflows, technologies that automate data acquisition may complement current abstraction processes by improving the timeliness and completeness of genomic information. Optical character recognition (OCR) converts scanned documents into machine-readable text and has demonstrated utility for extracting information from electronic health records (EHR) and other unstructured clinical documents [16–19]. Previous studies have combined OCR with natural language processing to improve automated abstraction of pathology reports and clinical documentation [17, 20]. However, relatively few studies have evaluated OCR for routine extraction of genomic biomarkers from clinical reports or examined its potential role in improving cancer registry data availability [21, 22]. Oncotype DX (OncoDx) is a validated 21-gene expression assay that yields a recurrence score (ranges 0–100) used to guide adjuvant chemotherapy decisions in early-stage breast cancer. OncoDx reports follow a highly standardized layout with a single, well-defined numeric result, making them an ideal and tractable first target for evaluating OCR performance.

This study evaluated and compared three open-source OCR approaches, Tesseract, EasyOCR, and a hybrid implementation, to determine which best enables automated extraction of OncoDx recurrence scores and improves the timeliness and quality of real-world oncology data. Using manually abstracted recurrence scores as the reference standard and cancer registry data as a real-world comparator, we assessed both the accuracy of automated extraction and factors associated with registry discordance.

## Methods

### Study design and open-source justification

We conducted a retrospective validation study using OncoDx reports stored as scanned PDFdocuments within Epic, a widely used EHR system, at a Midwestern U.S. health system between 2016 and 2022 [4]. Epic systems is a privately held information technology company, founded in 1979 and headquartered in Wisconsin, whose EHR software is the most widely used in the USA, running over 70% of health records of the National Cancer Institute Designated Cancer Centers [23, 24]. OncoDx is a 21-gene expression assay used to guide adjuvant treatment decisions for patients with early-stage, hormone receptor-positive, HER2-negative breast cancer. Recurrence scores range from 0 to 100 and were categorized according to the TAILORx trial as low (0–15), intermediate (16–25), and high (26–100) risk [25]. We selected Tesseract and EasyOCR because they are the two most widely used, actively maintained, and fully open-source OCR engines, and because they represent two distinct architectural approaches: Tesseract uses a mature long short-term memory-based recognition engine [26], whereas EasyOCR uses a modern deep-learning pipeline that couples Character Region Awareness for Text Detection (CRAFT) with neural sequence recognition [27, 28]. The two also differ in their computational requirements: EasyOCR benefits from GPU acceleration but can run on CPU, whereas Tesseract runs efficiently on CPU alone, a practical consideration for cancer registries and lower-resourced settings. We included the hybrid approach to test whether combining the two captures the complementary strengths of each. Throughout, we prioritized open-source tools because cost, transparency, and reproducibility are decisive factors for adoption by cancer registries and lower-resourced environments.

### Data extraction

**Fig. 1:**
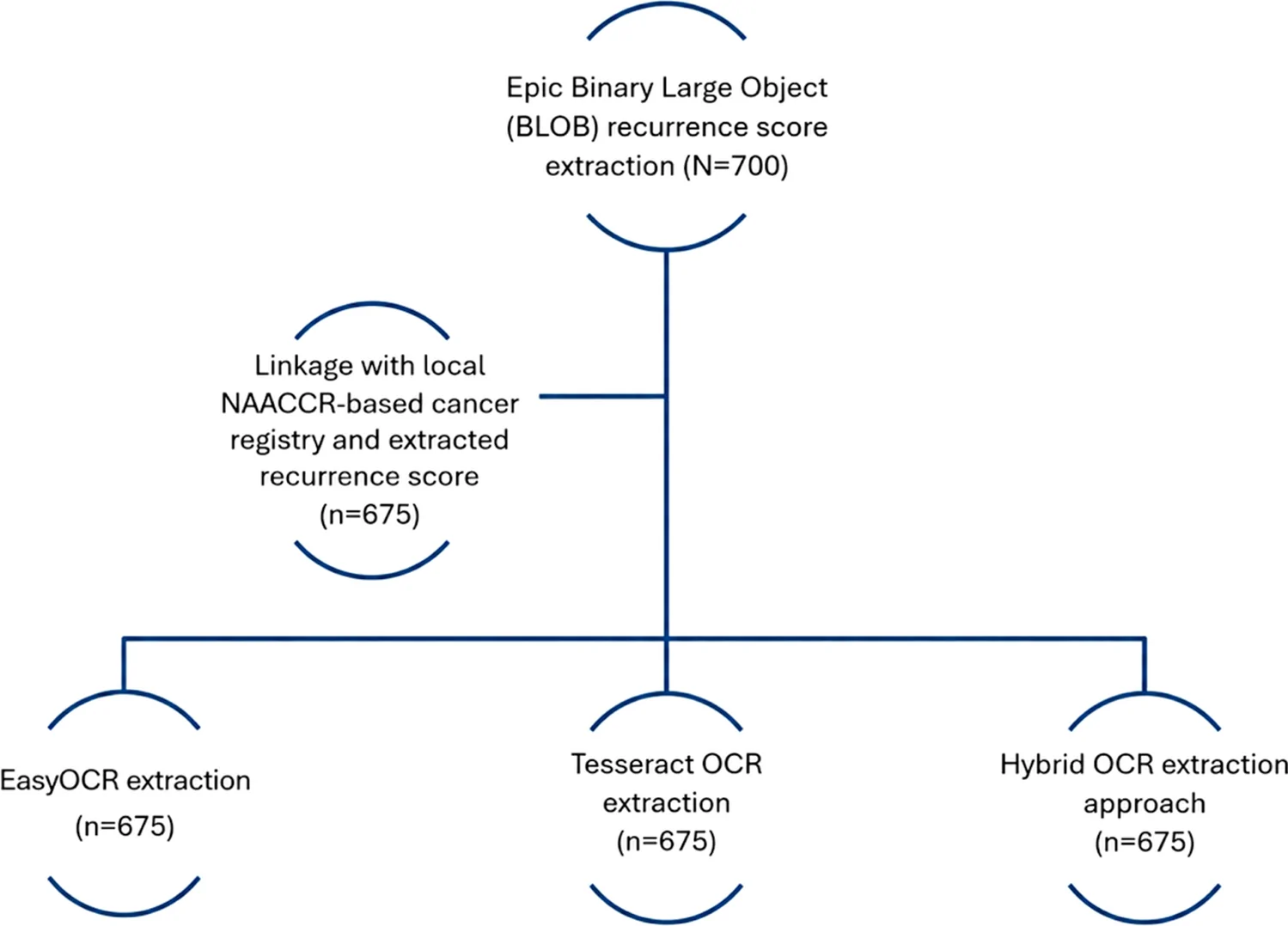
Diagram of genome data extraction from the electronic health record and cancer registry. *NAACCR* North American Association of Central Cancer Registried, *OCR* optical character recognition

Using the Binary Large Objects (BLOBs) functionality within Epic, 700 OncoDx reports were extracted from the EHR. OncoDx reports enter the Epic record as unstructured scanned/PDF documents rather than as discrete structured fields, and Epic stores such documents as BLOBs, the database representation used for large binary content. Because these documents were not available as structured, machine-readable data, OCR was required to extract the recurrence score. Following review, duplicate reports and documents without recurrence scores were excluded, resulting in 675 unique reports for analysis. Two independent reviewers (SM and QL) manually abstracted recurrence scores, over the course of 2 h, and served as the reference standard. Three OCR pipelines were evaluated: (1) EasyOCR [29], (2) Tesseract (Pytesseract 0.3.13 is a Python wrapper for Google’s Tesseract OCR engine) [30], and (3) a hybrid OCR (H-OCR) approach that primarily used EasyOCR while incorporating Tesseract for failed or ambiguous extractions. PDF pages were rendered at 300 DPI and converted to grayscale for image processing. Tesseract was configured using OCR Engine Mode 3 (OEM 3) and Page Segmentation Mode 10 (PSM 10), with Hough circle detection used to identify candidate circled scores. EasyOCR used CRAFT-based text detection with pretrained English-language recognition; images were scaled to 220% and Gaussian blurred, with adaptive thresholding and a circularity threshold > 0.7 used to identify candidate circled regions. EasyOCR was selected as the primary engine because its CRAFT-based text detection more reliably localized the circled recurrence score annotations in OncoDx reports, whereas Tesseract was retained as a fallback because of its complementary recognition capabilities and faster processing speed. Tesseract was triggered when EasyOCR returned no text, no detected circle, or no integer value. OCR-derived recurrence scores and corresponding cancer registry values were matched to manually abstracted scores for comparison (Fig. 1).

### Outcome measures and statistical analysis

The primary outcome was the accuracy of automated extraction compared with manual abstraction. OCR performance was evaluated using exact agreement, precision, recall, F1 score, OCR engine generated confidence scores, and processing time. Agreement was defined as exact concordance between OCR-derived and manually abstracted recurrence scores. Precision was defined as the proportion of recurrence scores identified by the OCR pipeline that were correct, whereas recall was defined as the proportion of true recurrence scores in the source documents that were successfully captured by the pipeline. High recall was particularly important because a false negative represents a recurrence score that would be missing from the resulting dataset. Performance was also assessed according to clinically relevant TAILORx risk categories (low, intermediate, and high).

OCR confidence scores were evaluated according to concordance with the manually abstracted reference standard. Confidence scores were retained in their native formats, with EasyOCR scores ranging from 0 to 1 and Tesseract scores ranging from 0 to 100 and were analyzed separately because confidence scores are engine specific and not directly comparable across OCR engines [31, 32]. For reports with detections across multiple pages, the recurrence score was determined by the most frequently extracted value, and confidence scores associated with that value were averaged across pages. When an OCR engine failed to return a usable extraction and no confidence score was available, the confidence score was assigned a value of 0 to represent extraction failure. Confidence distributions are reported as median and interquartile range (IQR) and were compared between concordant and discordant extractions using the Wilcoxon rank-sum test. Receiver operating characteristic (ROC) analysis was used to assess the ability of confidence scores to discriminate concordant from discordant extractions, with area under the ROC curve (AUC) and 95% confidence intervals calculated using the DeLong method [33]. For H-OCR, confidence discrimination was evaluated among Tesseract fallback extractions rather than pooling confidence scores across engines.

A secondary analysis evaluated factors associated with discordance between registry-reported and manually abstracted recurrence scores at the patient level among the 472 unique patients represented by the 675 reports. Multivariable logistic regression was performed with registry discordance (match vs. mismatch) as the dependent variable. Prespecified covariates included age, race, histology, tumor grade, tumor size, lymph node status, progesterone receptor (PR) status, chemotherapy, lymphovascular invasion, rural–urban commuting area (RUCA) classification, and Area Deprivation Index (ADI) category. Processing times were recorded for each OCR pipeline and compared with routine manual abstraction. All analyses were performed using Python 3.13 and R Studio 4.5.1. This study was determined to be exempt by the University of Minnesota Institutional Review Board.

## Results

### OCR performance

Across all 675 OncoDx reports, the H-OCR pipeline demonstrated the highest overall performance, achieving 97% agreement with manual abstraction, a precision of 0.997, recall of 0.972, and an F1 score of 0.984 (Table 1). Cancer registry abstraction achieved 91% agreement with manual review, with a precision of 0.992, recall of 0.973, and an F1 score of 0.983. EasyOCR achieved 93% agreement, with a precision of 0.981, recall of 0.956, and an F1 score of 0.968, whereas Tesseract achieved a precision of 0.990, recall of 0.934, and an F1 score of 0.961 (Table 1).

**Table 1:** Concordance metrics between open-source optical character recognition (OCR) methods, the hybrid OCR (H-OCR) extraction method, and manual extraction

	EasyOCR vs. manual	Tesseract versus manual	Hybrid OCR versus manual	Registry vs. manual
Metric				
Percentage positive	93	91	97	91
Precision	0.981	0.990	0.997	0.992
Recall	0.956	0.934	0.972	0.973
F1 Score	0.968	0.961	0.984	0.983

**Fig. 2:**
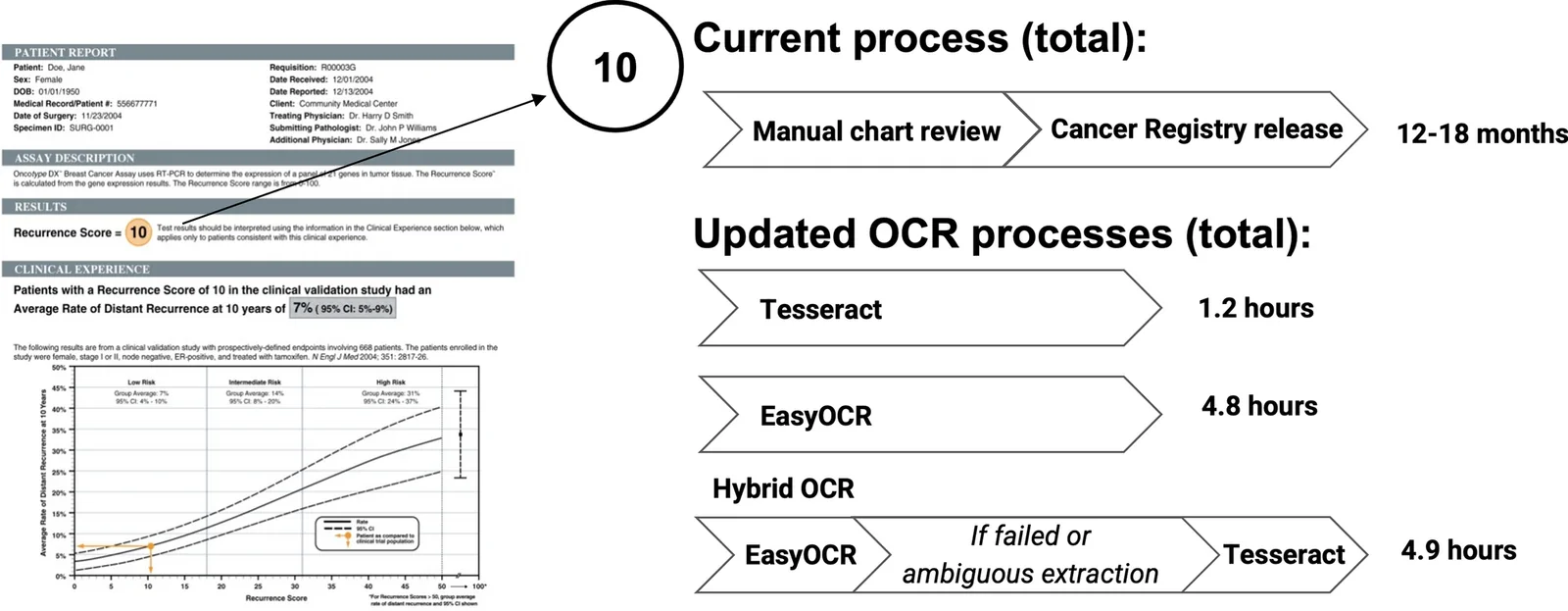
Anonymized oncotype report and current extraction time by modality and OCR software

The complete dataset of 675 OncoDx reports was processed in approximately 1.2 h using Tesseract, 4.8 h using EasyOCR, and 4.9 h using the H-OCR pipeline (Fig. 2).

### Factors associated with registry discordance

Patient-level analyses included 472 unique patients represented among the 675 reports (Table 2). Multivariable logistic regression demonstrated that discordance between registry-reported and manually abstracted OncoDx recurrence scores was largely independent of patient demographics, tumor characteristics, nodal status, treatment history, lymphovascular invasion, rurality, and neighborhood socioeconomic status. Unknown PR status was the only variable significantly associated with discordance (adjusted odds ratio [aOR] 2.88, 95% confidence interval [CI] 1.16–8.28; *p* = 0.032, Table 2). To address the clinical significance of the OCR discordances, we further characterized each discordant case according to whether the error shifted the patient across a TAILORx risk boundary, since only category-crossing errors can plausibly influence adjuvant treatment decisions. Among the 3% of files that were discordant with the H-OCR approach, most failed risk score recognitions were not risk category-crossing errors and showed no consistent pattern, although some appeared to stem from misread leading digits, missing leading digits, and poor fax or scan quality. A subset of these discordances altered TAILORx categorization. In cancer registry abstraction, eight cases classified as low-risk should have been intermediate risk and seven classified as intermediate-risk should have been high risk; with the H-OCR approach, six cases classified as low risk should have been high risk and three classified as low risk should have been intermediate risk. All category-crossing errors underestimated the true risk category.

**Table 2:** Demographic and tumor characteristics of patients with oncotype DX recurrence scores

	*n* = 472 (%)
Age	
18–49	80 (17)
50–64	227 (48)
65+	165 (35)
Race	
White	429 (91)
Other	43 (9)
RUCA	
Urban/metropolitan	424 (90)
Rural/nonmetropolitan	16 (3)
Rural/unknown	32 (7)
ADI category	
1	228 (48)
2	152 (32)
3 or 4	92 (20)
Grade	
Well	127 (27)
Moderate	260 (55)
Poor/undifferentiated	71 (15)
Missing	14 (3)
Tumor size	
< 2 cm	315 (67)
≥ 2 cm or unknown	157 (33)
Lymph node status	
Negative	376 (80)
Positive	86 (18)
Unknown	10 (2)
PR status^a^	
Negative	117 (25)
Positive	148 (31)
Unknown^a^	207 (44)
Chemotherapy	
No	377 (80)
Yes	95 (20)
Lymphovascular invasion	
Not Present	354 (75)
Present	97 (21)
Missing	21 (4)
Registry manual concordance	
Concordant	426 (90)
Discordant	46 (10)

^a^*Note:* In multivariable analysis, only unknown PR status was significantly associated with registry-manual discordance (adjusted OR (odds ratio) 2.88, 95% CI (confidence interval) 1.16–8.28; *p* = 0.032)

*Acronyms: RUCA* rural–urban commuting area, *ADI* area deprivation index, *PR* progesterone receptor

### Confidence score analysis

Confidence scores were higher for concordant than discordant extractions across both OCR engines (Table 3). For Tesseract, median confidence was 83.5 (IQR 76–90) for concordant and 45.0 (IQR 22–68.25) for discordant extractions (*p* < 0.001), with an AUC of 0.90 (95% CI 0.85–0.94). For EasyOCR, median confidence was 1.00 (IQR 0.92–1.00) for concordant and 0.00 (IQR 0.00–0.79) for discordant extractions (*p* < 0.001), with an AUC of 0.92 (95% CI 0.87–0.97). Within the H-OCR pipeline, 629 of 675 final extractions were generated by EasyOCR and were concordant, with a median confidence of 1.00 (IQR 0.92–1.00). The remaining 46 extractions used Tesseract as the fallback; confidence was higher for concordant than discordant extractions (median [IQR], 83.5 [76.5–91.5] vs. 51.0 [30.3–74.6], *p* < 0.001), with an AUC of 0.81 (95% CI 0.66–0.96) (Table 3).

**Table 3:** Discrimination of OCR confidence scores between concordant and discordant Oncotype DX recurrence score extractions

OCR methods	*n*	Discordant median [IQR]	Concordant median [IQR]	AUC (95% CI)	*p* value
Tesseract	675	45.0 [22–68.25]	83.5 [76–90]	0.90 (0.85–0.94)	<0.001
EasyOCR	675	0.00 [0.00–0.79]	1.00 [0.92–1.00]	0.92 (0.87–0.97)	<0.001
H-OCR (EasyOCR with Tesseract)	46	51.0 [30.3–74.6]	83.5 [76.5–91.5]	0.81 (0.66–0.96)	<0.001

*OCR* optical character recognition, *IQR* interquartile range [Q1–Q3], *AUC* area under the ROC curve, *CI* DeLong confidence interval

## Discussion

In this study, automated extraction of genomic biomarkers from unstructured clinical documents achieved higher agreement with manual abstraction than traditional cancer registry abstraction while substantially reducing the time required to process the complete cohort of 675 reports. The H-OCR pipeline achieved 97% agreement with manual review compared with 91% for registry abstraction while processing the entire dataset within hours rather than the months typically required for routine registry availability. Together, these findings demonstrate that automated genomic data extraction provides an accurate and scalable approach for accelerating genomic data capture and reducing the latency of real-world oncology data.

An important finding of this study was that registry discordance was largely independent of patient demographics, tumor characteristics, nodal status, treatment history, lymphovascular invasion, rurality, and neighborhood socioeconomic status. Unknown PR status was the only significant predictor of discordance, suggesting that remaining discrepancies are more likely attributable to incomplete documentation and abstraction challenges than to specific clinical subgroups. These findings suggest that improving cancer registry performance is not solely a matter of reducing reporting delays but also of improving the accuracy and completeness of genomic data capture. By extracting genomic assay results directly from source documents when they become available within the EHR, automated extraction has the potential to reduce both delays and information loss inherent to traditional registry abstraction workflows. Furthermore, because registry discordance was not associated with most patient or tumor characteristics, the benefits of automated genomic data capture may be broadly applicable across diverse patient populations [15].

The implications of automated genomic data capture extend beyond cancer registries. Timely and accurate genomic biomarkers are increasingly essential for comparative effectiveness research, quality improvement initiatives, precision oncology, and the development of artificial intelligence and machine learning applications using real-world oncology data [5, 6, 15]. Delayed or incomplete genomic information limits the ability of health systems to generate contemporary evidence from routine clinical practice. By enabling rapid and accurate capture of structured genomic data directly from clinical reports, automated extraction may help bridge the gap between clinical care and research while strengthening the infrastructure needed for cancer surveillance, real-world evidence generation, and learning health systems [14]. Because EasyOCR and Tesseract are open-source platforms that can be implemented within Health Insurance Portability and Accountability Act (HIPAA)-compliant environments, this approach also represents a practical and scalable solution for health systems seeking to modernize genomic data infrastructure. Ultimately, direct processing and integration of genomic biomarkers into structured EHR fields could further improve data accessibility and reduce reliance on chart review or document-based extraction. Until such integration is broadly implemented, OCR provides a practical approach for capturing genomic data that remain embedded in unstructured clinical documents.

To monitor cancer trends across the U.S. state population-based cancer registries collect and report data nationally. These registries use standardized formats to enable comparisons across geographic areas and time. Since 1997, the North American Association of Central Cancer Registries (NAACCR) has supported these efforts through an annual review program that certifies registries based on completeness, accuracy, and timeliness [8, 9]. Despite its importance, persistent challenges remain such as the lag in acquiring and processing cancer-related datasets, particularly genomic data, which limits the timeliness of insights that impact patient care in the era of personalized medicine [34, 35].

Cancer registry data, especially for genomic biomarkers, can be delayed by 12–18 months due to the need for manual chart abstraction from unstructured lab reports [36]. This process is not only time-consuming but also prone to human error and inconsistency [12]. A key barrier is the reliance on scanned outside documents stored as image files within the EHR. These documents are often disorganized, scanned in the order received rather than chronologically, and grouped under vague headings [13, 16, 17]. Our results indicate that this time lag gap can be diminished, especially for low-resourced cancer centers.

Previous studies have demonstrated the feasibility of OCR for extracting information from handwritten forms, pathology reports, and other EHR documents [16–20]. Rasmussen et al. developed an OCR pipeline for handwritten clinical forms that achieved high positive predictive value but limited sensitivity [16]. Hom et al. demonstrated that combining OCR with natural language processing substantially reduced manual abstraction time while maintaining excellent accuracy for oncology performance status extraction [17]. Laique et al. similarly reported successful automated extraction of quality metrics from colonoscopy reports using OCR and NLP [20]. Our findings extend these applications by demonstrating that automated extraction can accurately capture genomic biomarkers from routinely generated clinical reports while directly comparing performance with contemporary cancer registry abstraction. Rather than serving solely as an OCR performance study, our findings support automated genomic data extraction as a practical strategy for improving the timeliness and completeness of structured oncology data. OncoDx reports were an ideal and tractable first target for this evaluation: they follow a highly standardized layout built around a single, well-defined numeric result. Somatic next-generation sequencing (NGS) reports, by contrast, are far more heterogeneous in vendor, format, and content, an important and more challenging direction for future work.

For institutions seeking the highest accuracy, our results support the hybrid implementation, which achieved the best overall performance in our evaluation. Implementing the hybrid pipeline requires modestly more engineering effort than a single engine, running two OCR engines and applying an arbitration step, but it requires no specialized hardware or proprietary software. For settings where simplicity, speed, or limited computing resources are paramount, standalone Tesseract is a reasonable alternative given its high precision and substantially faster runtime, with a modest trade-off in recall. Conversely, EasyOCR was selected as the primary engine in the hybrid pipeline because of its higher recall (0.956 vs. 0.934). A reversed Tesseract-primary configuration may warrant evaluation in settings where processing speed is prioritized. Our hybrid pipeline did not incorporate the engine-level confidence scores produced by EasyOCR and Tesseract into its arbitration logic; fallback to Tesseract was triggered when EasyOCR returned no text, no detected circle, or no integer value. However, our confidence score analysis demonstrated strong discrimination between concordant and discordant extractions for both EasyOCR and Tesseract, supporting confidence as an additional measure of extraction reliability [31, 32]. These findings suggest that future implementation of engine-specific confidence thresholds could help flag uncertain extractions for targeted human review and further reduce the risk of clinically consequential misclassification.

Several limitations should be considered. First, this study was conducted at a single health system, and OCR performance may differ across institutions with different document formats, scanning practices, or EHR systems. Second, only OncoDx reports were evaluated, and future studies should determine whether similar performance can be achieved across other commercially available genomic assays. Third, although automated extraction substantially reduced the time required to process the complete study cohort, we did not prospectively evaluate implementation into routine cancer registry workflows or quantify its impact on reporting timeliness, abstraction workload, registry completeness, or downstream clinical decision-making. Future multi-institutional implementation studies are needed to determine how automated extraction performs across diverse health systems and how best to integrate these approaches into existing cancer registry workflows.

In conclusion, automated genomic data extraction from unstructured clinical documents provides a practical and scalable approach for reducing genomic data latency in oncology. Compared with traditional cancer registry abstraction, automated extraction achieved higher agreement with manual review while making structured genomic information available substantially earlier. As genomic testing becomes increasingly integrated into routine cancer care, automated genomic data capture has the potential to modernize cancer registry infrastructure, accelerate real-world evidence generation, and support the growing demand for timely, high-quality oncology data in precision medicine.

## Data Availability

The datasets analyzed during the current study are not publicly available because they contain protected health information and institutional clinical records. De-identified data may be available from the corresponding author upon reasonable request and with approval from the institution.
